# Pediatric pilonidal sinus disease: Recurrence rates of different age groups compared to adults

**DOI:** 10.1016/j.sopen.2025.01.001

**Published:** 2025-01-07

**Authors:** Dietrich Doll, Susanne Haas, Ida Kaad Faurschou, Theo Hackmann, Henrike Heitmann, Myriam Braun-Münker, Christina Oetzmann von Sochaczewski

**Affiliations:** a Klinik für Proktochirurgie und Pilonidalsinus, St. Marienhospital, Vechta, Germany; b Pilonidal Disease Center, Department of Surgery, Regionshospitalet Randers, Denmark; c Department of Clinical Medicine, Aarhus Universitetshospital and Aarhus Universitet, Aarhus, Denmark; d Department of Clinical Epidemiology, Aarhus Universitetshospital and Aarhus Universitet, Aarhus, Denmark; e Vechtaer Institut für Forschungsförderung, Vechta, Germany; f Fachbereich Lebensmitteltechnologie, Hochschule Fulda, Fulda, Germany; g Chirurgische Klinik, Universitätsklinikum Bonn, Bonn, Germany

**Keywords:** Pilonidal sinus, Therapy, Recurrence rate, Children, Adolescents

## Abstract

**Background:**

Pilonidal sinus disease uncommon in pre-pubertal children. The preferred treatment for pediatric pilonidal sinus patients remains unclear. A growing body of evidence indicates that pediatric pilonidal sinus disease recurs earlier than in adults. We therefore aimed to investigate recurrence rates and the recurrence rates of different surgical approaches.

**Methods:**

Some 1217 studies on pilonidal sinus disease, encompassing 134,663 patients were eligible. From them, 5807 pediatric patients were identified. Recurrence rates were compared between adults and children.

**Results:**

Pediatric pilonidal sinus patients have a higher 5-year recurrence rate compared to adults (46 % vs. 11.5 %; *p* < 0.0001). The subgroup of individuals aged 16–18 years appears to experience recurrences 12 months earlier than those below the age of 16. 46.4 % of all pediatric recurrences occur within 5 years.

**Conclusions:**

Pediatric pilonidal sinus disease seems to follow a different course in terms of recurrence rate compared to adults with a substantially increased probability of developing recurrent pilonidal sinus disease within the first five years after surgery. Due to the limited evidence base, especially in terms of the surgical approach, additional data is required to gain a more detailed insight into the matter and to improve surgical care for children and adolescents.

## Highlights

• Pilonidal sinus recurrences quadruple in minors compared to adults after five years.
• Recurrences seem to occur earlier between 16 and 18 years compared to younger children.
• The 5-year recurrence rate of pilonidal sinus disease in minors is 46.4 %.

## Introduction

Pilonidal sinus disease is rare in pre-pubertal children [1,2], rendering it a topic of limited interest within the pediatric surgical community in the past [3]. Documented recurrence rates after surgery in children and adolescents varied between 0 and 42 % between studies irrespective of the surgical approach that was used [4]. This prompted *Golladay & Wagner* to remark, “Incision or excision and packaging, marsupialization, excision with closure, injection, destruction of the tract, irradiation, and various plastic and rotational procedures and grafts have all had less than desirable outcomes.” [5] An assessment frequently encountered in the literature or as *Mattei* pointed it “[…] in general, when there are many operations described to fix a problem it usually means they are all suboptimal.” [6] The “ideal” surgical approach has been investigated in a systematic review of pediatric studies, which did not identify a procedure that could be beneficial compared to others in terms of recurrence [7], and resulted in the development of treatment algorithms that favored repeated minimally-invasive interventions, the aspect of time to recurrence had not emerged until recently. *Maasewerd* and co-workers [8] noted that several studies [[9], [10], [11], [12]] reported median time to recurrence from 2.9 months [13], although that might rather be treatment failure than recurrence, to 12 months [14]. A finding that has been corroborated in a review, which was able to demonstrate that the majority of recurrences in the pediatric population occurs in the first postoperative year [4]. This observation prompted us to examine the temporal distribution of recurrences after surgical treatment for pilonidal sinus disease using all data available from the literature.

## Methods

**Fig. 1:**
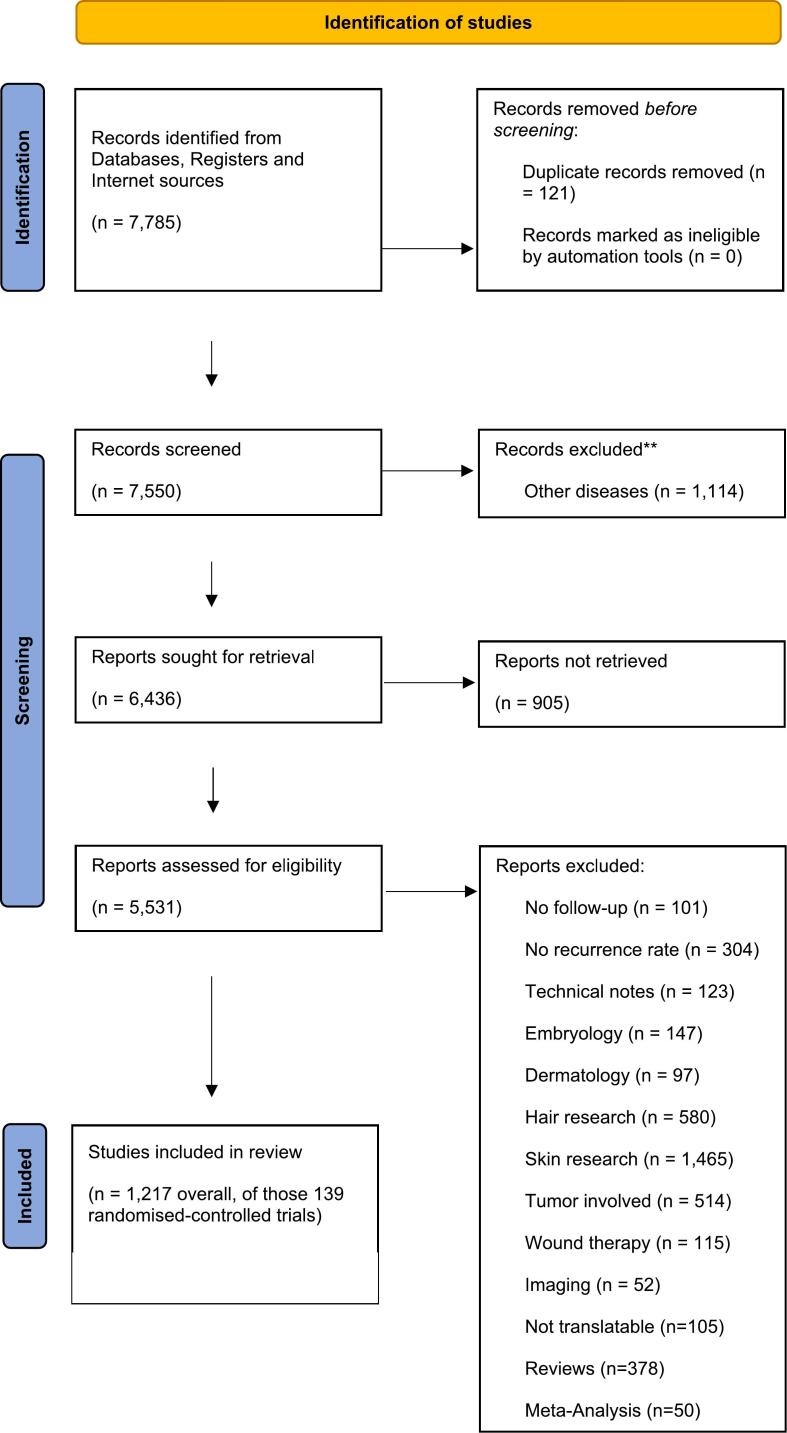
PRISMA flow chart.

Data was curated following an established method [[15], [16], [17], [18]], resulting in the following PRISMA flow diagram [19] results (Fig. 1).

### Search strategy and study selection criteria

To compile a comprehensive database related to pilonidal sinus disease (PSD), a systematic search for the NCBI Medical Subject Heading (MeSH) term “pilonid*,” as well as the combination of “dermoid” AND “cyst” in the following databases was conducted: MEDLINE, PubMed, PubMed Central, Scopus, Ovid, Embase, and Cochrane Central Register of Controlled Trials (CENTRAL). Additionally, searches using these terms in Google, Google Scholar, and ResearchGate were performed. This was supplemented by searching the references cited in national and international guidelines. References listed in the bibliographies of all documents retrieved through these searches were scrutinized. The retrieved documents encompassed a range of study types, including randomized and non-randomized trials, prospective and retrospective studies, and observational studies, such as cohort, case-control, cross-sectional studies, and case reports, published from 1833 to 2023.

Three researchers (DD, HH, and TH) reviewed the retrieved documents for adherence to the inclusion criteria, which required the documentation of definitive treatment, recurrence, and the duration of follow-up. Disagreements between them were solved via consensus, if this could not be reached, a fourth researcher (IKF) cast a decisive vote. Reports published in English, French, German, Italian, and Spanish were considered, as well as publications in other languages if they provided an English abstract detailing the definitive treatment, recurrence, and follow-up time. Authors were contacted via email or ResearchGate for English translations of their surgical approaches, recurrence data, and follow-up times if necessary. Exclusion criteria were pilonidal sinus disease occurring in locations other than the pre-sacral region, involvement of neoplasia, and duplicate publication of data by the same author. Studies lacking any component of the minimal data set (comprising definitive treatment strategy, recurrence, and follow-up time) were also excluded. Previous meta-analysis reports and review articles were excluded, but their reference lists were scrutinized for potential additions to the evidence. Additionally, unpublished data presented in review articles were considered.

### Data collection, extraction, and quality assessment

All studies were analyzed and documented on paper. Transcribed data were then entered into a Microsoft Excel spreadsheet (Version 2016, Microsoft, Redmond, United States of America) and subject to verification to ensure accuracy. Each specific therapeutic strategy reported in a paper was assigned to a separate line, with columns including citation details, the number of patients included, therapeutic procedures, reported follow-up times, study details, and recurrence data. Since the statistical measures used for reporting follow-up times varied among studies, with mean and median reports being treated as equivalent due to the clustering of disease incidence in young adults, we used the center of the reported range for data with a range of follow-up times. For data that included minimum follow-up times, the values were incorporated as reported. We assessed individual studies for consistency in their methods and reported results to minimize potential bias in data synthesis. A subgroup analysis of prospective randomized controlled trials was conducted separately to ensure consistency with the overall set of studies. The reported recurrence rates in each study were then linked to the study's respective follow-up time, which was defined as mean, median, center of range, or minimum. To facilitate comparison across all studies, single patients were statistically simulated, with each study participant representing a data sample containing recurrence, follow-up time, and therapeutic procedure. Certain information, such as gender ratios, could not be included as it was only available cumulatively in the majority of studies.

### Statistical analyses

For statistical analysis and visualization, we utilized the R statistical software package (version 4.3.1) within the R-studio framework (version 23.6.1.524). Statistical significance was determined at *p* < 0.05 [Log-Rank-test], and all tests were conducted in a two-tailed setup. To assess the recurrence-free outcome over time, we employed survival analysis according to Kaplan-Meier, including pointwise 95 % confidence intervals, as implemented in the R-package ‘survival’ (version 3.5.5). The package ‘survminer’ (version 0.4.9) was used for the graphical display of the data. These analyses were performed for each defined therapeutic procedure and age groups as outlined, and results were presented as the percentage of recurrence-free outcomes with their corresponding 95 % confidence interval. The horizontal axes of the plots included the numbers of patients within specific follow-up intervals: 0–12, 12–24, 24–60, 60–120, and > 120 months. If specific data were unavailable for an interval, linear interpolation was applied to estimate recurrence-free outcomes based on the two nearest observed follow-up times. To provide a comprehensive assessment of recurrence and a rational foundation for selecting treatment procedures, we evaluated data in the manner of a classical meta-analysis of randomized-controlled trials, and we also conducted an analysis that integrated non-randomized controlled trials. Due to the stepwise nature of the Kaplan-Meier curves, small discrepancies between the plotted and tabulated values may occur.

To assess potential heterogeneity in the study outcomes, we utilized Cochrane analysis and calculated Ι [2]. The articles were grouped based on procedures and follow-up time intervals, as utilized for the Kaplan-Meier curve plots. Separate analyses were conducted for studies derived from randomized controlled trials and for the entire set of studies. For computing Cochrane's Qs to evaluate heterogeneity, recurrence data as reported in the corresponding studies were weighted by the respective numbers of study participants. A Χ^2^ test was employed to assess the significance of heterogeneity, and Ι^2^ was calculated to complement the Χ^2^ test results.

## Results

**Fig. 2:**
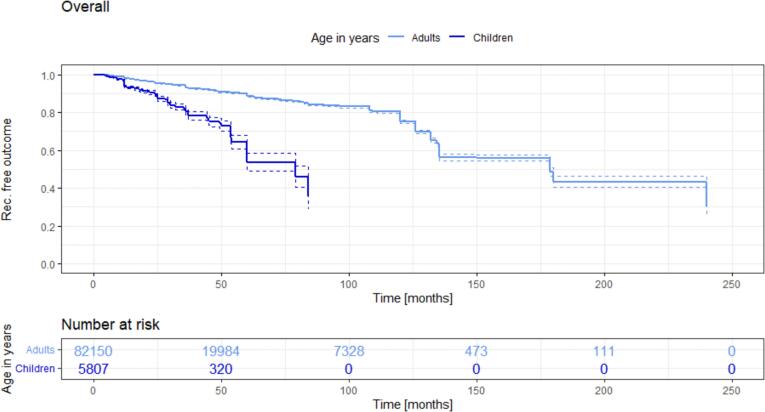
Recurrence free outcome in adults (light blue line) versus children and adolescents (<18 years of age) since surgery [in months]. (For interpretation of the references to colour in this figure legend, the reader is referred to the web version of this article.)

In total, 1217 studies with 134,663 patients, published from 1833 to 2023, were included and analyzed (Fig. 1). In these studies, 5807 pediatric patients were identified. Kaplan-Meier analysis was used to investigate the recurrence-free outcomes across all studies, stratified by age groups: those with a mean age both above and below 18 years (Fig. 2).

In adults, the recurrence rate demonstrates a mean annual increase around 2 % over the follow-up period. Specifically, the 5-year recurrence rate stands at 11.5 %, and the 10-year recurrence rate at 24.9 %. The trajectory of recurrence-free outcomes in studies involving participants with a mean age below 18 years (dark blue line) exhibits an earlier and continuing decline: At 24 months after surgery, the recurrence rate reaches 10.5 %. After 60 months, an extremely high recurrence rate of 46.4 % is observed in the pediatric group (*p* < 0.0001 compared to adults). Nearly every second pediatric patient, within this group, will encounter a recurrence of their pilonidal sinus disease. A similar recurrence rate is only reached within the adult group a decade later, manifesting as a 44.1 % recurrence rate at 178 months after the initial surgery.

**Fig. 3:**
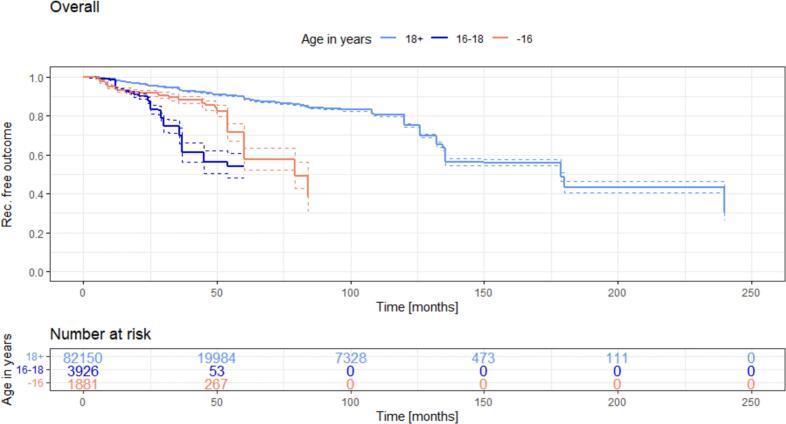
Recurrence free outcome in adults above age 18 (light blue line) versus children above 16 years (deep blue line) and versus children below 16 years (red line). (For interpretation of the references to colour in this figure legend, the reader is referred to the web version of this article.)

In a subgroup analysis, the pediatric population was divided into patients below 16 years and patients of 16 to18 years of age and compared to the adult population (Fig. 3).

While both pediatric groups appear similar initially, they begin to diverge after 25 months post-surgery: The group of <16 years appears to closely follow the gradual incline of recurrences observed in the adult group, the group of 16 to 18 years of age experiences a relevant increase in recurrence rate, reaching 30.4 % at 36 post-surgery (vs. 11 % in adults, *p* = 0.0001). This difference persists 5 years post-surgery (*p* = 0.0001). Consequently, pediatric recurrences are four times more frequent than adult recurrences five years post-surgery. The highest recurrence rate occurs in the age group spanning 16 to 18 years at 50 months postoperatively. It may be possible that the cohort of 16–18 years children experiences earlier recurrences that the group of below 16 years, but this cannot be deduced from the data.

## Discussion

Pediatric pilonidal sinus disease had long been an issue of lesser interest for the surgical community [3], but gained more attention due to rising incidences [20]. Systematic reviews primarily focused on the “the” surgical procedure of choice and included either only pediatric studies [7] or a mixture of pediatric and adult studies [21], despite the Cochrane Review stating that results from adult studies should not be applied to children and adolescents [22]. It has been noted before that in many studies of pediatric pilonidal sinus disease recurrences were common and occurred in up to 41 % of included cases [23], but their temporal distribution was not recognized to be an issue until recently [4,8]. We therefore aimed to assess the recurrence dynamics of pilonidal sinus disease in children and adolescents, because every recurrence in them negatively impacts the formative phase of life.

The relevance of the recurrence dynamics originates from the effect on treatment choices. In the literature, the surgical approaches are rather diverse and span from epilation alone [23] to lateralizing flap procedures [24]. As recurrences are common, irrespective of the chosen surgical approach [4,7,8,21,24], it is necessary to be aware of their temporal distribution in order to guide follow-up and treatment. Due to the supposedly increased recurrence rates, treatment algorithms have been proposed that focus on repeated minimally-invasive procedures to reduce the negative impact on the patient's daily life activities [21,25,26].

We were able to show what has already been suspected elsewhere [4,8,27] that many recurrences in pediatric pilonidal sinus disease occur earlier compared to adults, but that the recurrences were not confined to the first year after surgery, but increases thereafter to almost thrice the rate in adults three years after surgery. That information is crucial, because promising reports using a minimally-invasive approach had limited follow-up times of only some months [23,[28], [29], [30], [31]] or slightly above one year [32], which would thus have missed many of the recurrences that were still to come.

With a median follow-up of 1.9 years, the duration of follow-up in a study using Gips' technique [33] is higher [34], but does still not reach the duration of follow-up in which the curves in our study started to diverge between children and adolescents at the verge of adulthood. Another issue with Gips' technique, which has been criticized for its non-reproducibility of its good results in adults outside the originally describing institution [35], even at surgical departments affiliated to the same university [[36], [37], [38]], seems to be that this pattern also occurs in children and adolescents. *Dreznik* and co-workers reported excellent results in them using Gips' technique [34], but the results were much less favorable at another center affiliated to the same university [39]. One aspect that might be used to explain these differences could be the learning curve, as it has been suggested for a modification of Gips' technique [40], but currently there is no data on that matter.

Lack of available data also is a relevant limitation of this study. We were only able to use crude recurrence rates irrespective of the used surgical techniques or other potentially modifying factors such as body mass index [[41], [42], [43]], age [9], comorbidities [44], or sex [9,43], although none of them did consistently reproduce in other studies [8,32]. This precludes a more differentiated analysis of our results, because accounting for these factors would require an individual-patient meta-analysis for which the data are not available at all.

However, this highlights another limitation of our study that is again related to a lack of data: The small number of persons under surveillance with larger follow-ups. This might have exaggerated our results with excessively large recurrence rates in adolescents at the verge to adulthood, but without additional data, this is mere speculation. One would have expected to see more than one register study [9] as a reaction to rising incidences of pilonidal sinus disease [20,45] or other real-world data. For example, more focus on surgical procedures would require additional knowledge on treatment approaches used from urban centers to rural clinics. Such data is occasionally available for adults from some countries [[46], [47], [48], [49], [50]], but only from Turkey for children and adolescents [51]. Nonetheless, we felt it would be preferable to accept the higher heterogeneity associated with using crude recurrence rates and patients included over a large time span in order to gain at least some insights into the recurrence dynamics of pediatric pilonidal sinus disease beyond short-term follow-up.

In order to obtain more detailed and less heterogeneous results, more high-quality data is necessary, which currently is scarce for pediatric pilonidal sinus disease and currently limited to the randomized-controlled trial on postoperative laser-epilation [52], which demonstrated a substantial reduction in recurrence rate following laser-epilation [53], although this effect might not serve all population groups equally [54].

## Conclusion

Pediatric and adolescent pilonidal sinus patients seem to experience recurrences more often and in a different temporal distribution, recurrences occur earlier, compared to adults, especially in adolescents close to adulthood. The limited evidence base precludes differentiation of recurrence rates between surgical approaches or to identify factors associated with recurrences. Additional high-quality data, both from trials and real-world evidence, is necessary to identify modifiable or driving factors of this difference to adults and to adapt patient care to these challenges.

## CRediT authorship contribution statement

**Dietrich Doll:** Writing – original draft, Supervision, Project administration, Methodology, Investigation, Formal analysis, Data curation, Conceptualization. **Susanne Haas:** Writing – original draft, Supervision, Project administration, Investigation, Conceptualization. **Ida Kaad Faurschou:** Writing – review & editing, Methodology, Formal analysis. **Theo Hackmann:** Writing – review & editing, Visualization, Investigation, Formal analysis, Data curation. **Henrike Heitmann:** Writing – review & editing, Software, Methodology, Investigation, Formal analysis, Data curation. **Myriam Braun-Münker:** Writing – review & editing, Validation, Supervision, Software, Methodology. **Christina Oetzmann von Sochaczewski:** Writing – review & editing, Writing – original draft, Investigation, Conceptualization.

## Ethics statement

Ethical review and approval was not required for the study on human participants in accordance with the local legislation and institutional requirements. Written informed consent for participation was not required for this study in accordance with the national legislation and the institutional requirements.

## Funding statement

This research did not receive any specific grant from funding agencies in the public, commercial, or not-for-profit sectors.

## Declaration of competing interest

The authors declare that they have no known competing financial interests or personal relationships that could have appeared to influence the work reported in this paper.
